# The Impact of Diffusion-Weighted MRI on the Definition of Gross Tumor Volume in Radiotherapy of Non-Small-Cell Lung Cancer

**DOI:** 10.1371/journal.pone.0162816

**Published:** 2016-09-09

**Authors:** Jochen Fleckenstein, Michael Jelden, Stephanie Kremp, Philippe Jagoda, Jonas Stroeder, Fadi Khreish, Samer Ezziddin, Arno Buecker, Christian Rübe, Guenther K. Schneider

**Affiliations:** 1 Department of Radiotherapy and Radiation Oncology, Saarland University Medical Center, Homburg, Germany; 2 Department of Diagnostic and Interventional Radiology, Saarland University Medical Center, Homburg, Germany; 3 Department of Nuclear Medicine, Saarland University Medical Center, Homburg, Germany; Generalitat Valenciana, SPAIN

## Abstract

**Objective:**

The study was designed to evaluate diffusion-weighted magnetic resonance imaging (DWI) vs. PET-CT of the thorax in the determination of gross tumor volume (GTV) in radiotherapy planning of non-small-cell lung cancer (NSCLC).

**Materials and Methods:**

Eligible patients with NSCLC who were supposed to receive definitive radio(chemo)therapy were prospectively recruited. For MRI, a respiratory gated T2-weighted sequence in axial orientation and non-gated DWI (b = 0, 800, 1,400 and apparent diffusion coefficient map [ADC]) were acquired on a 1.5 Tesla scanner. Primary tumors were delineated on FDG-PET/CT (stGTV) and DWI images (dwGTV). The definition of stGTV was based on the CT and visually adapted to the FDG-PET component if indicated (e.g., in atelectasis). For DWI, dwGTV was visually determined and adjusted for anatomical plausibility on T2w sequences. Beside a statistical comparison of stGTV and dwGTB, spatial agreement was determined with the “Hausdorff-Distance” (HD) and the “Dice Similarity Coefficient” (DSC).

**Results:**

Fifteen patients (one patient with two synchronous NSCLC) were evaluated. For 16 primary tumors with UICC stages I (n = 4), II (n = 3), IIIA (n = 2) and IIIB (n = 7) mean values for dwGTV were significantly larger than those of stGTV (76.6 ± 84.5 ml vs. 66.6 ± 75.2 ml, p<0.01). The correlation of stGTV and dwGTV was highly significant (r = 0.995, p<0.001). Yet, some considerable volume deviations between these two methods were observed (median 27.5%, range 0.4–52.1%). An acceptable agreement between dwGTV and stGTV regarding the spatial extent of primary tumors was found (average HD: 2.25 ± 0.7 mm; DC 0.68 ± 0.09).

**Conclusion:**

The overall level of agreement between PET-CT and MRI based GTV definition is acceptable. Tumor volumes may differ considerably in single cases. DWI-derived GTVs are significantly, yet modestly, larger than their PET-CT based counterparts. Prospective studies to assess the safety and efficacy of DWI-based radiotherapy planning in NSCLC are warranted.

## Introduction

Radiotherapy (in combination with chemotherapy) is the treatment of choice for inoperable locally advanced NSCLC, and–with PET based staging–median survival averages 21 months [1]. For inoperable early stage NSCLC, stereotactic body radiotherapy (SBRT) can offer a high curative potential with local control rates amounting to 89% [2]. Modern, highly conformal radiotherapy techniques, such as SBRT and intensity modulated radiotherapy (IMRT), allow for dose escalation or can decrease normal tissue toxicity [3,4]. The success of high-precision radiotherapy is determined not only by an adequate control of daily patient positioning during the treatment course (safeguarded by ‘image guided radiotherapy’ (IGRT)) but, first and foremost, by an accurate target volume definition. As FDG-PET fulfills the demand for high diagnostic accuracy in the staging of NSCLC, it can enhance the quality of treatment planning in NSCLC significantly [5–9]. However, FDG-PET based treatment planning in NSCLC involves some unresolved issues. The spatial resolution of FDG-PET images is limited to 5 mm, which makes it prone to inaccuracies in GTV definition. Even though useful recommendations exist with respect to FDG-PET based definition of the GTV of the primary tumor, which take into account alternative contouring methods (e.g. visual, (semi-)automatic threshold based), no ‘gold standard’ has to date been established [9]. Also, dedicated FDG-PET scanners for radiotherapy planning are lacking in most centers, so that concerns can be raised about reproducible patient positioning and about timeliness when the initial staging FDG-PET is being used.

Due to the technical evolution of MRI-scanners, MRI based functional imaging (specifically diffusion weighted imaging (DWI)) has gained a considerable degree of diagnostic potential in lung cancer. Meanwhile, a number of studies were published which suggest an equivalent diagnostic performance for DWI as compared to FDG-PET with respect to T and N staging [10–13]. The growing availability of MRI scanners which can generate diffusion weighted thoracic images, their cost-effectiveness as compared to FDG-PET, and short scan protocols make DWI an attractive candidate to be validated in NSCLC radiotherapy planning. In the present study we aimed to evaluate volumetric deviations of the primary tumor as delineated on PET-CT versus diffusion weighted MRI and thereby sought to gain information about the nature of DWI based GTV definition.

## Material and Methods

### Study population and inclusion criteria

Eligible patients were at least 18 years old and had a pathologically confirmed diagnosis of non-metastasized NSCLC (UICC stages I–III), had no contraindications to MRI, did not receive any previous antitumoral therapy and were scheduled to receive–depending on tumor stage–either SBRT or definitive radiotherapy with concurrent chemotherapy. All patients signed informed consent before study inclusion. This prospective study was conducted in accordance with the Helsinki declaration and was approved by the local ethics committee (*Ärztekammer des Saarlandes*).

### Acquisition of CT, FDG-PET and MRI images

For the acquisition of the planning CT and MRI, patients were immobilized in the supine position with both arms above their head by using the WingSTEP^™^ system (Elekta, Stockholm, Sweden). Laser markings were used to enhance reproducibility of the positioning. The planning CT was a Philips BigBore^™^ 120 kV scanner (Philips Medical Systems, Amsterdam, The Netherlands). Patients received iodinated intravenous contrast medium. The slice thickness was 3 mm, images were acquired during free shallow breathing.

FDG-PET images were obtained from the diagnostic FDG-PET staging procedure, but no dedicated FDG-PET for radiotherapy planning was acquired. Yet, only patients who had received their FDG-PET in the Department of Nuclear Medicine of Saarland University Medical Center were recruited, i.e. FDG-PET scanning was institutionally standardized and specifically prepared to be used for additional radiotherapy planning with the same immobilization device being available. A Siemens Biograph^™^ PET/CT scanner was used (Siemens, Erlangen, Germany), ^18^F-FDG-PET acquisitions took place 90 minutes after injection.

All MRI examinations were performed using the same 1.5T MRI scanner (Magnetom Aera, Siemens, Erlangen, Germany). Patients were placed in an MRI-compatible immobilization device (WingSTEP) to enable identical positioning as in the CT.

A Half Fourier Acquisition Single Shot Turbo Spin Echo (HASTE) sequence (TE = 91 ms, TR = 1000 ms, Flip-angle = 125°, averages = 1, slice thickness 5 mm, FOV = 285x380 mm, matrix = 320x192) was acquired in transversal and coronal planes. To generate diffusion-weighted images a single-shot echo planar diffusion-weighted sequence with Stejskal-Tanner diffusion encoding scheme with use of an inversion recovery for fat saturation (TR = 15400 ms, TE = 75 ms, TI = 180 ms, PAT factor of 2, 3-scan trace (averaged), averages = 4, slice thickness 5 mm, FOV = 309x380 mm, matrix = 208x128 (interpolated to 208x256), no gap) was acquired. The real voxel size of the sequence is 1.5 x 3 x 5 mm^3^. Two b-values at b = 0 and b = 800 s/mm2 were acquired. Fusion Images were composed of the HASTE and the DWI. ADC maps and additional high b-value images at b = 1400 s/mm^2^ are calculated automatically by the scanner software, based on linear signal decay. Both HASTE and DWI sequences were acquired with the patient freely breathing and, in addition, fusion images were composed of the HASTE and the DWI.

**Fig 1:**
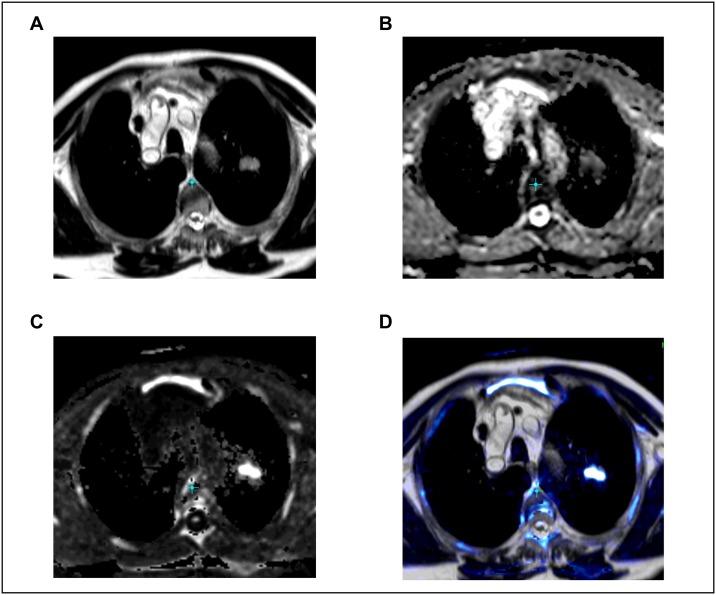
MRI sequences of a patient with stage T1b NSCLC in the left upper lobe. **A**. T2 HASTE sequence (non-gated). **B.** ADC-map. **C.** DWI (calculated b-value of 1400). **D.** Fusion of T2 and DWI, color represents diffusion restriction.

In addition, a respiratory gated T2-weighted sequence (TE = 106 m, TR = 3692 ms, Flip-angle = 160°, averages = 2, 3 mm slice thickness, FOV = 277x370 mm, matrix = 384x202) was acquired in a transversal plane. An example for the acquired MRI sequences is given in Fig 1.

### Contouring of the primary tumor

For comparison two GTVs of the primary tumor (involved lymph nodes were excluded) were generated in each patient: a “standard” GTV (stGTV), which was based on the PET-CT scans, and the GTV based on diffusion weighted MRI (dwGTV). The stGTV was primarily delineated on the planning CT by a senior radiation oncologist and a senior radiologist. To generate stGTV the use of soft tissue and lung windows (with standardized settings) as well as the assessment in the axial, sagittal and coronary plane were mandatory. The recommendations for generating the target volume as outlined by the European Organisation for Research and Treatment of Cancer (EORTC) were meticulously followed [14]. In a second step, the stGTV was assessed and–if indicated–visually modified on the basis of the anatomically registered FDG-PET. Yet, no FDG-PET based adjustments were allowed in case of peripheral NSCLC, which was surrounded by lung tissue and could hence be safely delineated on CT images alone. Also, the FDG-PET image set was to be disregarded, if it had not been performed within 4 weeks prior to the planning CT–as based on the recommendations of a consensus report of the *International Atomic Energy Agency (IAEA)* [9].

The delineation of the dwGTV was performed by two experienced senior radiologists on the registered T2 weighted and diffusion weighted MR image sets. The performing radiologists were blinded for the corresponding stGTV of the same patient. The dwGTV was visually contoured and secondarily checked and–if necessary–adjusted for anatomical plausibility on the corresponding T2 weighted sequence.

The contouring and automated volumetric measurement of the GTV of the primary tumors (GTV-PT) on PET-CT and MRI image sets was performed with the Pinnacle^3™^ V9.0-9-6 treatment planning software (Philips Medical Systems, Amsterdam, The Netherlands).

### Hausdorff-distance and Dice similarity coefficient as measures of spatial agreement

Two different methods have proven to be suitable to assess spatial concordance between two image or volume sets, both of which were used for the present analysis. One measure is the *Hausdorff-distance (HD)*, which reflects spatial deviations between a reference and model volume structure and is based on a ‘nearest neighbor’ measurement of two reference points of both structures. An in depth description of the mathematical foundation of HD was provided by Huttenlocher et al. [15]. Both the maximum and average HD were calculated. The other applied measure of agreement is the *Dice similarity coefficient (DSC)*, which is an index to quantify a spatial overlap between two segmented volume sets *A* and *B* and is defined as *DSC(A*,*B) = 2(A∩B)/(A+B)*, where *∩* is the intersection of both volume sets. Thus, DSC values of 0 vs. 1 indicate no vs. complete spatial agreement. For further reference we recommend the paper of Zou et al. [16]. Both HD and DSC values were generated with an open source software for biomedical research ‘3D Slicer’ [17]. Therefore, GTV-PTs as delineated on PET-CT and DWI were manually registered according to anatomical plausibility using rigid image fusion with image translation (without rotation) in three planes (anterior-posterior, lateral and cranio-caudal direction). To adjust for a ‘baseline shift’ of primary tumors in the different image sets, image fusion aimed at the best possible overlay of primary tumors, whereas fusion of adjacent bony structures was secondary.

### Statistical analysis

Data rows were analyzed for statistical differences by using a paired sample *t* test (in case of normal distribution). The relationship between PET-CT and DWI-based volumes was assessed using linear regression analysis and Pearson’s correlation coefficient. Bland-Altman plots were used to show the variation of measured volume differences between PET-CT and DWI.

## Results

### Patients’ and disease characteristics

Fifteen patients (3 female, 12 male) with histologically proven NSCLC were included in the study between July 2013 and September 2015. The median age was 65.5 years (range 55–79 years). One patient had two synchronous lesions of NSCLC (in both upper lobes), which were evaluated separately in volumetric assessment. Of the initially recruited sixteen patients one patient (case 11, Table 1) was secondarily excluded from the study because the extent of the primary tumor could not be measured on PET-CT due to concomitant postobstructive pneumonia. TNM stages and the localization of the primary tumor are shown in Table 1. The distribution of UICC-stages and histology was as follows: IA (n = 3), IB (n = 1), IIA (n = 2), IIB (n = 1), IIIA (n = 2) and IIIB (n = 7); squamous cell cancer (n = 11), adenocarcinoma (n = 4, whereof two were present in one patient) and ‘not otherwise specified’ (n = 2). In 3 patients with early stages (one of them with two lesions) stereotactic body radiotherapy was indicated while definitive radiochemotherapy was planned for 12 patients with locally advanced stages.

**Table 1:** Patient characteristics, measured tumor volumes for all 16 tumors in 15 patients.

Case^a^	TNM^b^	Localization	FDG-PET/CT	DWI-MRI^c^	p
			GTV (ml)	GTV (ml)	
1	T1bN0M0	Peripherally, left lower lobe	23.5	27.6	
2	T2aN2M0	Centrally, right lower lobe	33.5	40.1	
3	T4N2M0	Centrally, left upper lobe	58.9	88.0	
4	T4N2M0	Centrally, right upper lobe	65.7	65.4	
5	T1bN1M0	Peripherally, left upper lobe	7.1	5.2	
6	T4N2M0	Centrally, left upper lobe	286.1	317.9	
7	T4N2M0	Centrally, right upper lobe	103.6	113.8	
8	T3N0M0	Centrally, middle lobe	95.1	106.4	
9	T4N2M0	Centrally, right hilum	102.8	129.9	
10	T4N3M0	Centrally, left hilum	46.1	59.1	
12A	T1bN0M0	Peripherally, right upper lobe	6.6	4.1	
12B	T1aN0M0	Peripherally, left upper lobe	3.0	4.5	
13	T4N2M0	Centrally, left upper lobe	175.7	197.9	
14	T4N0M0	Centrally, left hilum	16.3	24.8	
15	T2aN1M0	Centrally, left lower lobe	18.7	10.9	
16	T2aN0M0	Centrally, left lower lobe	22.9	30.8	
mean ± SD			66.6 ± 75.2	76.6 ± 84.5	0.004
Median			39.8	49.6	
Range			3.0–286.1	4.1–317.9	

GTV, gross tumor volume;

^a^One patient (case 11) was disregarded because of concomitant inflammatory changes of lung tissue, which precluded reliable contouring of the gross tumor volume on FDG-PET/CT-images;

^b^TNM staging was based on FDG-PET/CT-extent and–if available–histologic mediastinal lymph node findings;

^c^the volumes are based on DWI-MRI sequences but were additionally checked and–if necessary–adjusted for anatomical plausibility on the corresponding T2-MRI-sequences; SD, standard deviation.

### Agreement between PET-CT and DWI in thoracic staging

PET-CT and DWI-based UICC-staging as well as T-stages were in complete agreement for all patients. Nevertheless, discrepancies were observed in N-staging: in 3 patients with multilevel lymph node involvement as diagnosed by PET-CT (but not pathologically confirmed) only single level lymph node involvement was visible with DWI, which would have resulted in downstaging from N3 to N2 in one of these patients.

### Volumetric analysis of the primary tumor: PET-CT vs. DWI

The median interval between the acquisition of the planning CT and the MRI was 8 days (range 5–12 days). Importantly, stGTV could be delineated safely on CT alone in 12 out of 16 primary tumors and was not modified after the review of the FDG-PET. In 4 patients with concomitant atelectases, stGTV was primarily determined with the aid of FDG-PET.

**Fig 2:**
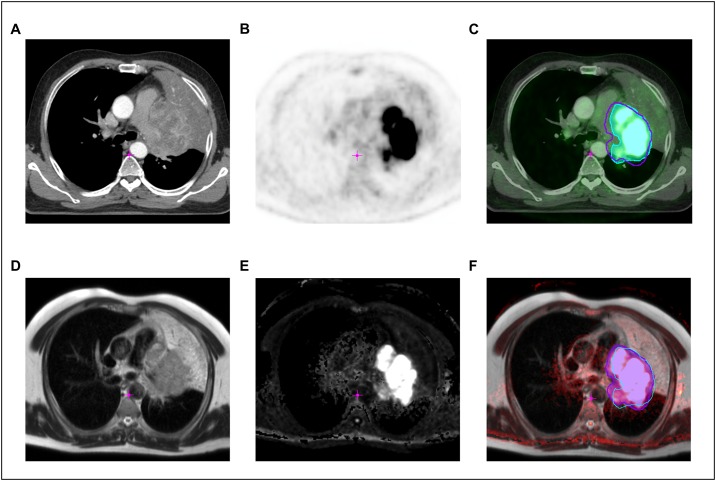
Example of PET-CT vs. MR including GTV contours in a patient with a large adenocarcinoma in the left hilum with concomitant atelectasis of the upper lobe. **A.** Axial slice of the contrast enhanced planning CT. **B.** FDG-PET-scan. **C.** Anatomically registered FDG-PET/CT. The turquois contour (colorwash) indicates the FDG-PET-based delineation of the GTV (stGTV), the corresponding DWI-based GTV (dwGTV) is overlaid as a purple line. **D.** T2-TSE axial. **E.** DWI-sequence (calculated b-value of 1400). **F.** Anatomically registered T2/DWI. The purple contour (colorwash) indicates the DWI-based delineation of the GTV (dwGTV), the corresponding FDG-PET-based GTV (stGTV) is shown as a turqois line.

**Fig 3:**
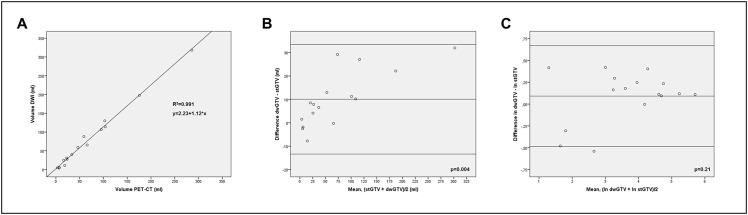
Scatter plots and Bland-Altman analysis of tumor volume **A.** Linear regression analysis with fitted line. *p* = 0.005. **B.** Bland-Altman analysis showing upper and lower limits of agreement (mean volume difference ± 2 standard deviations) between stGTV (x-axis) and dwGTV (y-axis). The p-value indicates a significant dependence of measured volume differences from the average tumor size, as tested with linear regression analysis. **C.** Bland-Altman analysis showing upper and lower limits of agreement (mean volume difference ± 2 standard deviations) between stGTV (x-axis) and dwGTV (y-axis) in logarithmic scale. In logarithmic form, no significant dependence of measured volume differences from the average tumor size was observed (tested with linear regression analysis).

PET-CT and DWI-derived volumes are depicted in Table 1 for all patients’ primary tumors. The mean percentage difference between stGTV and dwGTV volumes was 30.9% (± 21.1 sd (standard deviation)) and the median difference was 27.5%, range 0.4–52.1%) as referenced to the smaller volume, i.e. either stGTV or dwGTV, whichever applied. It was relatively larger for T1-T2 tumors (n = 7) as opposed to T3-T4 tumors (n = 9) (41.7% ± 20.1 vs. 22.5% ± 18.2, p = 0.07). The GTV of the primary tumor was larger in DWI than in PET-CT in 12 cases and smaller in 4 cases. On average, dwGTV was significantly larger than stGTV (76.6 ml ± 84.5 vs. 66.6 ± 75.2, p = 0.004). In 4 patients with lobar (n = 3) or segmental (n = 1) atelectases, stGTV and dwGTV were in good agreement (an example is given in Fig 2). In spite of the volume deviations a high correlation was found between stGTV and dwGTV (Fig 3A). As analyzed with Bland-Altman plots a high concordance between PET-CT and DWI volumetric measurements was found: as expected, the absolute volume differences between PET-CT and DWI significantly increased with growing tumor volumes as validated by linear regression analysis (Fig 3B). Nevertheless, the huge range of tumor volumes had to be taken into account (3.0–286.1 ml, as measured with PET-CT). To adjust for this scaling effect, Bland-Altman analysis was additionally performed with logarithmic volume data as recommended by Bland and Altman [18]. In that logarithmic form, no significant dependence of the volumetric differences between PET-CT and DWI from tumor size was found as tested with linear regression analysis (Fig 3C).

### Spatial agreement–Hausdorff distance and Dice similarity coefficient

As evaluated on registered PET-CT and DWI image set pairs, maximum HD was 16.3 ± 8.1 mm, average HD was 2.25 ± 0.7 mm, and DSC was 0.67 ± 0.09. These data show an acceptable overall agreement, even though the maximum HD hints at considerable incongruence in subunits of the compared GTVs.

## Discussion

For radiotherapy of NSCLC the exact delineation of the primary tumor is of vital importance. In terms of volumetric comparison, PET-CT and DWI based (independently contoured) GTVs were highly correlated. Yet, DWI based GTVs were significantly larger (10.0 ml, on average) than those which were generated on PET-CT. The median intraindividual deviation of stGTV and dwGTV amounted to 27.5%. This seemingly large difference has to be put into relative perspective as it equals the radial expansion of a sphere containing 66.6 ml (the average stGTV) by 2.1 mm. Spatial concordance was acceptable between these two methods, as indicated by the mean DSC of 0.67 and the average HD of 2.25 mm. However, maximum HD values exceeded 15 mm in small subunits, which–under real planning conditions–would hence not have been covered by a standard planning target volume (as derived from the respective smaller GTV, i.e. stGTV or dwGTV, and subsequently expanded by usually less than 15 mm).

As indicated by the logarithmic Bland-Altman plot (Fig 3C) the overall degree of similarity between DWI and PET-CT based contouring is relatively high and robust throughout the large spread of examined volumes ranging from 3.0–286.1 ml (as measured on PET-CT). Thus, we assume that DWI based GTV delineation may be just as accurate as PET-CT. Schaefer et al. [19] provided valuable volumetric measurements of primary tumors in 15 patients with NSCLC based on FDG-PET as compared with the same tumors, which were subsequently resected and laminated. Their pathologic volume was calculated by means of an elaborate procedure. In that study, a high correlation between volumes obtained from FDG-PET and pathology was reported, but the pathological volume was overestimated by FDG-PET by 32.5 ± 26.5%. In the context of those data, the findings of the present study may imply that DWI based GTV assessment may also overestimate the “true” tumor volume by a small margin.

The results have to be interpreted in the light of the specific planning conditions: no rigorous head-to-head comparison of DWI vs. FDG-PET volumes was performed. Instead, a comparison between DWI (supported by a T2 sequence) and ‘standard’ approach of generating the GTV on the basis of the planning CT with the integration of the diagnostic FDG-PET. As a limitation, tumor motion was not explicitly quantified for the analyzed tumors, which may at least partially have contributed to the observed volumetric differences.

Given the fact that to date no clinical data exist, what benefits could be expected by integrating DWI in the process of treatment planning in NSCLC? Evidence suggests that under the prerequisite of validated scan protocols, DWI and FDG-PET are equally accurate in mediastinal and hilar staging of NSCLC, as the meta-analysis of Wu et al. concludes [20]. Everitt et al. [21] stressed the importance of the use of an up-to-date FDG-PET for treatment planning: in a cohort of 17 NSCLC patients who received two technically identical FDG-PET scans prior to radiotherapy with a median interscan period of 24 days, the volume surrounded by a standardized uptake value of at least 3 increased by 63.4% and a probability of upstaging of 32% was reported. We believe that DWI may emerge as a more versatile and available tool than FDG-PET to be used for treatment planning because it can be obtained in most high volume radiology departments with scan times of approximately 20 minutes and is therefore cost effective. In anticipation of trial protocols designed to evaluate the clinical outcome of DWI based treatment planning, we believe we have quantified the “margin of change” when the primary tumors are delineated with DWI as opposed to PET-CT. A deeper integration of DWI into NSCLC treatment planning demands a significant amount of further research. For DWI, alternative contouring methods like visual contouring as opposed to ADC-threshold based volume delineation need future attention as do the impact of interobserver variability and most importantly its validation in clinical use, issues which have already been addressed extensively in regard to FDG-PET based treatment planning, thus manifesting its current lead.

## Conclusions

Altogether, the independent PET-CT and MRI based GTV definition of the primary tumor yields an acceptable degree of similarity. Nevertheless, tumor volumes may differ considerably in single cases. DWI-derived GTVs are significantly, yet modestly, larger than their PET-CT based counterparts. Prospective studies to assess the safety and efficacy of DWI-based radiotherapy planning in NSCLC are warranted. For the time being, diffusion-weighted MRI remains a promising diagnostic means, which may increasingly be used in radiotherapy planning of NSCLC.
